# Impact of lockdown on COVID-19 prevalence and mortality during 2020 pandemic: observational analysis of 27 countries

**DOI:** 10.1186/s40001-020-00456-9

**Published:** 2020-11-10

**Authors:** Sultan Ayoub Meo, Abdulelah Adnan Abukhalaf, Ali Abdullah Alomar, Faris Jamal AlMutairi, Adnan Mahmood Usmani, David C. Klonoff

**Affiliations:** 1 Department of Physiology, College of Medicine, King Saud University, PO Box 2925, Riyadh, 11461 Saudi Arabia; 2 Diabetes Research Institute, Mills-Peninsula Medical Center, San Mateo, California USA

**Keywords:** COVID 19, Lockdown, Quarantine, Prevalence, Mortality, Epidemiological trends

## Abstract

**Background:**

This study aimed to assess the impact of 15 days before, 15 days during, and 15 days after the lockdown on the trends in the prevalence and mortality in 27 countries during COVID-19 pandemic.

**Methods:**

Twenty-seven countries were randomly selected from the different continents. The information on the trends in the prevalence and mortality due to COVID-19 pandemic in 27 countries was obtained from World Health Organization and lockdown data were obtained from concerned countries and their ministries. The impact of lockdown for 15 days before, 15 days during, and 15 days after the lockdown on the prevalence and mortality due to the COVID-19 pandemic in 27 countries was analyzed.

**Results:**

The findings showed that 15 days after the lockdown there was a trend toward a decline, but no significant decline in the mean prevalence and mean mortality rate due to the COVID-19 pandemic compared to 15 days before, and 15 days during the lockdown in 27 countries. The mean growth factor for number of cases was 1.18 and for mortality rate was 1.16.

**Conclusions:**

The findings indicate that 15 days after the lockdown, daily cases of COVID-19 and the growth factor of the disease showed a declined trend, but there was no significant decline in the prevalence and mortality.

## Background

The “Severe Acute Respiratory Syndrome Coronavirus 2 (SARS-CoV-2) also known as COVID-19 pandemic”, originated in Wuhan, China, spread globally and has caused destruction of human health, lives, and economies [1]. As of July 02, 2020, this disease has involved 216 countries and has infected 10,533,038 people with a mortality rate of 512,842 (4.86%) [3].

The median incubation period for COVID-19 is 5.1 days, and can be up to 14 days. The incubation period of COVID-19 is important to understand in establishing, monitoring, surveillance and control of the disease [4]. The severe contagious nature of COVID-19 has led to an unhealthy situation across the world. The worldwide population is 7.6 billion [5], and the major part of the population, approximately 3.9 billion people, has been under lockdown and quarantined in their homes at some point [6]. The lockdown and quarantine polices have been implemented by many nations to minimize the spread of this disease and bring it under control. The lockdown includes isolation at homes, travel restrictions, and termination of all public events. Modern lockdown strategies have been enforced all over the world in order to prevent the COVID-19 infection from spreading even further [7].

Currently, no established vaccines and pharmacological interventions are known to effectively cure or prevent a COVID-19 infection, and public health measures such as lockdown, quarantine, and social distancing appear to be the only ways to curb the outbreak. Lockdown and quarantine can either be implemented on a voluntary basis, or if deemed necessary, can be legally imposed by the authorities, and may be applied at individual or community levels [8, 9]. The home quarantine, when scientifically and adequately applied and exercised according to modern principles and practices, can be an effective method for preventing the spread of contagious diseases, such as COVID 19. Globally, many countries have extended a lockdown, quarantine period for over 2 months. There are great concerns about the effectiveness and risks of long-term implementation of a lockdown and or quarantine [10]. Keeping in view the rapid transmission of COVID-19 cases globally, the present study aimed to investigate the impact of lockdowns for 15 days before, 15 days during and 15 days afterward on international epidemiological trends in the prevalence and mortality of COVID-19 cases.

## Materials and methods

The present observational study was conducted in the “Department of Physiology, College of Medicine, King Saud University” during the period of May–June 2020. The data on the trends in the prevalence and mortality due to COVID-19 outbreak were acquired from World Health Organization [3]. The daily reports on COVID-19 published by the World Health Organization were carefully reviewed and data were collected [3]. The lockdown data were obtained from concerned countries and their ministries however, populations of the countries were obtained from the world bank [5]. Twenty-seven countries were randomly selected from the different continents. The information on the trends in the prevalence and mortality due to COVID-19 outbreak in these 27 countries was obtained from the World Health Organization [3] and lockdown data were obtained from countries and their allied organizations.

We defined the growth factor as a ratio by which a quantity multiplies itself over time; it equals daily cases divided by cases on the preceding day. A growth factor of more than 1.0, indicates an increasing pattern of prevalence, whereas values between below 1.0 show a declining pattern [11]. This number is equivalent to the velocity of a car: positive means the car is moving forward and negative means the car is moving backward. We defined the growth factor per day as the change in growth factor from one day to the next. A positive growth factor indicates exponential growth in the number of cases and a negative growth factor per day indicates exponential decay in the number of new cases. This number is equivalent to the acceleration of a car: positive means the car is accelerating (speeding up) and its velocity is increasing more and more per time period and negative means the car is decelerating (slowing down) and its velocity is decreasing more and more per time period. A negative growth rate per day means an epidemic is coming under control because in that case, the number of new cases each day will be decreasing and heading in a direction toward no new cases in a day.

The data were carefully recorded and examined because the pandemic has been evolving, with the numbers changing daily. We analyzed the impact of lockdown on the “prevalence and mortality due to COVID-19” outbreak in 27 countries by establishing an association between the numbers, 15 days before, 15 days during and 15 days after the end of the lockdown period.

## Statistical analysis

The data were recorded and analyzed, and the results were expressed in numbers and percentages. The mean and SEM of prevalence and mortality cases was calculated. The growth factor, by which quantity multiplies itself over time; daily cases divided by cases on the previous day was calculated. A *p*-value < 0.05 was considered significant.

## Results

The worldwide number of cases and deaths due to COVID-19 are presented in Table 1. The impact of the lockdown on the epidemiological trends is presented in Tables 2 and 3. These tables contain the mean number of cases and mean number of deaths, respectively, at 15 days before, 15 days during, and 15 days after the lockdown in 27 countries and correlations were established. Worldwide, the COVID-19 infection has involved 216 countries and territories, and has infected 10,533,038 people with a mortality rate of 512,842 (4.86%) (Table 1) during the period December 29, 2019 to July 02, 2020 (Table I). The majority of these coronavirus cases, was reported from the American Region 5,317,792(50.48%), European Region 2,747,810 (26.08%), Eastern Mediterranean Region 1,096,565 (10.41%), South-East Asia Region 833,735 (7.91%), African Region 318,432 (3.02%) and Western Pacific Region 218,704 (2.07%) (Table 1).

**Table 1:** Worldwide number of laboratory-confirmed cases and deaths due to Covid-2019 pandemic

Number	Total	(%)	Number of deaths	(%)
Total number of cases	10,533,038	100	512,842	4.86
Western Pacific Region	218,704	2.07	7471	0.07
European Region	2,747,810	26.08	198,489	1.88
South-East Asia Region	833,735	7.91	22,861	0.21
Easter Mediterranean Region	1,096,565	10.41	25,286	0.24
American Region	5,317,792	50.48	252,396	2.39
African Region	318,432	3.02	10,272	0.09

World Health Organization, data updated on July 02, 2020^3^

**Table 2:** COVID-19 pandemic: mean number of cases 15 days before, 15 days during and 15 days after the lockdown in 27 countries

Date of lockdown	Country	Cases 15 days before the lockdown (mean ± SEM)	Cases 15 days during the lockdown (mean ± SEM)	Cases 15 days after the lockdown (mean ± SEM)	*p* value
March 24, 2020	Algeria	1.60 ± 0.61	22.80 ± 5.51	100.33 ± 17.05	0.0001
March 20, 2020	Argentina	6.40 ± 2.22	69.07 ± 14.26	101.27 ± 15.32	0.0003
March 23, 2020	Australia	68.53 ± 18.32	313.13 ± 41.68	53.53 ± 10.85	0.0001
February 25, 2020	Bahrain	0.00	7.20 ± 2.04	19.20 ± 3.46	0.0002
March 12, 2020	Belgium	17.73 ± 6.48	311.33 ± 56.92	1362.13 ± 73.78	0.0001
March 18, 2020	Chile	10.40 ± 5.33	172.13 ± 44.15	385.07 ± 19.89	0.0001
January 23, 2020	China	36.92 ± 14.25	1841.13 ± 31	3012.07 ± 902.45	0..0001
March 24, 2020	Colombia	13.00 ± 4.55	85.93 ± 13.23	171.33 ± 15.30	0.0001
April 1, 2020	Cuba	11.07 ± 4.30	39.73 ± 3.94	45.80 ± 3.04	0.0001
March 15, 2020	Djibouti	0.00	2.00 ± 0.82	22.47 ± 6.24	0.0002
March 25, 2020	Egypt	20.47 ± 3.99	72.27 ± 10.15	168.80 ± 20.10	0.0001
March 17, 2020	France	431.53 ± 113.51	2493.60 ± 346.68	3578.53 ± 312.89	0.0001
March 23, 2020	Germany	1377.87 ± 491.51	4928.53 ± 311.99	3097.93 ± 286.01	0.0001
March 30, 2020	Ghana	9.07 ± 5.25	28.60 ± 11.01	73.67 ± 23.93	0.0638
March 25, 2020	India	31.67 ± 8.61	317.00 ± 65.47	1147.47 ± 78.56	0.0001
March 9, 2020	Italy	490.93 ± 110.73	3450.87 ± 479.02	4777.27 ± 229.39	0.0001
March 24, 2020	Nepal	0.00	0.53 ± 0.24	2.40 ± 1.06	0.0003
March 30, 2020	Nigeria	4.20 ± 1.78	17.20 ± 3.41	66.27 ± 30.06	0.0428
April 1, 2020	Pakistan	120.80 ± 14.67	274.87 ± 41.01	712.80 ± 110.83	0.0001
March 15, 2020	Philippines	4.36 ± 1.56	67.40 ± 24.63	245.13 ± 51.65	0.0001
March 24, 2020	Republic of the Congo	0.44 ± 0.24	2.73 ± 1.54	9.13 ± 3.28	0.0676
March 31, 2020	Russian Federation	100.00 ± 24.60	1304.53 ± 227.84	5467.20 ± 820.22	0.0001
March 25, 2020	Saudi Arabia	50.13 ± 15.41	144.33 ± 12.05	787.67 ± 90.40	0.0001
March 26, 2020	South Africa	36.47 ± 11.92	86.07 ± 25.22	144.21 ± 19.76	0.0001
March 14, 2020	Spain	280.40 ± 97.02	3988.53 ± 690.01	6251.40 ± 381.66	0.0001
March 23, 2020	United Kingdom	320.53 ± 80.20	2852.80 ± 377.51	5162.40 ± 299.56	0.0001
March 17, 2020	United States of America	117.86 ± 38.08	9261.73 ± 2208.38	29,391.40 ± 880.14	0.0001

**Table 3:** COVID-19 pandemic: mean number of deaths 15 days before, 15 days during and 15 days after the lockdown in 27 countries

Date of lockdown	Country	Deaths 15 days before lockdown (mean ± SEM)	Deaths 15 days during lockdown (mean ± SEM)	Deaths 15 days after lockdown (mean ± SEM)	*p* value
March 24, 2020	Algeria	0.07 ± 0.07	1.60 ± 0.43	17.80 ± 3.07	0.0002
March 20, 2020	Argentina	0.13 ± 0.09	1.93 ± 0.48	6.13 ± 0.90	0.0003
March 23, 2020	Australia	0.33 ± 0.16	2.13 ± 0.48	2.13 ± 0.42	0.0001
February 25, 2020	Bahrain	0.00	0.00	0.27 ± 0.12	N/A
March 12, 2020	Belgium	0.00	11.87 ± 4.34	186.60 ± 31.89	0.0001
March 18, 2020	Chile	0.00	0.80 ± 0.30	5.93 ± 0.77	0.0001
January 23, 2020	China	0.75 ± 0.28	37.00 ± 5.55	114.07 ± 12.78	0.2228
March 24, 2020	Colombia	0.13 ± 0.13	2.20 ± 0.57	10.00 ± 1.25	0.0001
April 1, 2020	Cuba	0.27 ± 0.12	1.13 ± 0.29	2.47 ± 0.35	0.0001
March 15, 2020	Djibouti	0.00	0.00	0.13 ± 0.09	N/A
March 25, 2020	Egypt	1.20 ± 0.40	5.00 ± 0.66	12.73 ± 1.07	0.0001
March 17, 2020	France	9.73 ± 2.99	191.27 ± 33.04	908.80 ± 118.09	0.0001
March 23, 2020	Germany	4.47 ± 2.09	91.13 ± 13.48	218.13 ± 17.41	0.0001
March 30, 2020	Ghana	0.27 ± 0.15	0.27 ± 0.15	0.53 ± 0.34	0.7231
March 25, 2020	India	0.60 ± 0.27	9.33 ± 2.69	36.60 ± 1.86	0.0001
March 9, 2020	Italy	24.27 ± 8.74	340.67 ± 57.47	736.80 ± 29.16	0.0001
March 24, 2020	Nepal	0.00	0.00	0.00	N/A
March 30, 2020	Nigeria	0.07 ± 0.07	0.60 ± 0.24	2.00 ± 0.94	0.027
April 1, 2020	Pakistan	1.67 ± 0.50	5.47 ± 0.93	± 18.53 ± 2.79	0.0001
March 15, 2020	Philippines	0.07 ± 0.07	4.40 ± 1.59	17.13 ± 3.84	0.0001
March 24, 2020	Republic of Congo	0.00	0.33 ± 0.19	0.07 ± 0.07	0.1885
March 31, 2020	Russian Federation	0.60 ± 0.29	10.73 ± 1.98	58.33 ± 9.00	0.0001
March 25, 2020	Saudi Arabia	0.07 ± 0.07	2.67 ± 0.53	5.53 ± 0.34	0.0001
March 26, 2020	South Africa	0.00	1.20 ± 0.35	4.07 ± 1.13	0.0013
March 14, 2020	Spain	8.00 ± 3.27	315.87 ± 67.56	752.13 ± 33.08	0.0001
March 23, 2020	The United Kingdom	15.40 ± 4.86	313.40 ± 66.70	797.60 ± 35.45	0.0001
March 17, 2020	United States of America	3.87 ± 1.18	157.13 ± 42.25	1534.73 ± 113.21	0.0001

**Fig. 1:**
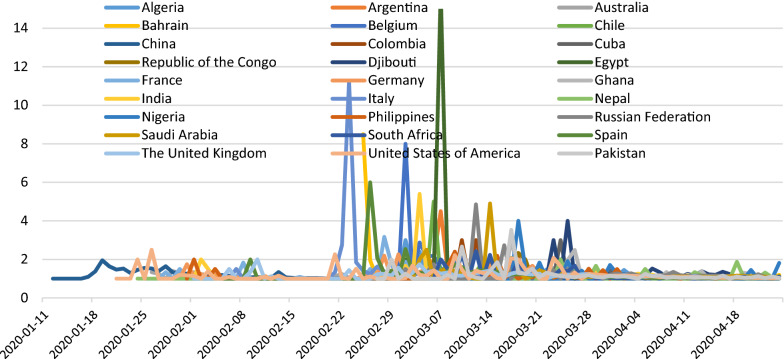
Growth factor of number of cases 15 days before, 15 days during and 15 days after lockdown

**Fig. 2:**
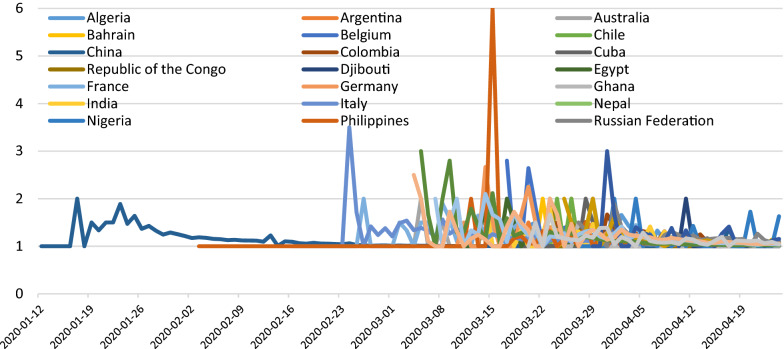
Growth factor of number of deaths 15 days before, 15 days during and 15 days after the lockdown

Regarding the impact of lockdown on the prevalence and mortality of the COVID-19 outbreak in 27 countries, we found that 15 days after the lockdown there was no decline in the mean prevalence and the mean number of daily deaths due to COVID-19 compared to 15 days before and 15 days during the lockdown (Tables 2 and 3). However, the growth rate in the number of new daily cases of COVID-19 per day (Fig.1) and growth rate in the number of deaths per day (Fig. 2) attributed to COVID-19 each showed a positive but declining trend 15 days after the lockdown period in most of the sampled countries. This data indicated a negative growth factor per day during the 15 days following the lockdown for new daily cases and for deaths per day. The growth factor per day results for new cases and deaths for each country are presented in Figs. 1 and 2. The change in growth rates and growth rates per day are expressed first as the difference between two time periods: pre-lockdown vs. lockdown and second between two time periods: lockdown vs. post-lockdown. The tables and figures illustrate that post-lockdown time periods, there was a declining rate of change per day for most of the 27 individual countries and for the entire 27-country cohort (Figs. 1 and 2).

Regarding the mean prevalence of COVID-19 cases 15 days before, 15 days during and 15 days after lockdown, we found that the mean numbers of cases in all these 27 countries increased and there was no significant impact of lockdown on the prevalence of COVID-19 cases (Tables 2 and 3). We quantified growth rate for new cases of COVID-19. The mean growth rate for number of cases on a daily basis was 1.18 and for mortality rate was 1.16. It was identified that, 15 days after the lockdown, the growth factor of the number of new daily cases was and the growth factor of daily deaths in these selected countries worldwide was decreased after the lockdown period.

## Discussion

The COVID-19 pandemic is an emerging worldwide health problem which has infected millions of people globally^2^. The notion of lockdown was linked with the incubation period of COVID-19, which is a median of 5.1 days and can be up to 14 days [4]. The lockdown policies have been enforced in many countries to reduce the spread of COVID-19 [6]. For a treatment to be successful at keeping an epidemic under control it must first cause the rate of growth per day to become negative and the growth rate to the growth rate per day to become negative fall to below 1.0, which would indicate an exponential decay in the number of cases.

In this study, we investigated the impact of 15 days before, 15 days during and 15 days after lockdown on the epidemiological trends in the prevalence and mortality because of the outbreak of novel coronavirus SARS-COV-2. We found that 15 days after the international lockdown there was no significant decline in the mean prevalence and mean mortality rate due to COVID-19 compared to 15 days before and 15 days during the lockdown in 27 countries. However, daily cases of COVID-19 and growth rates showed declining trends by the end of the 15 days after the lockdown period, leading to a critically important negative growth rate per day by the end of the lockdown period for both new daily cases and deaths. This negative growth rate per day in these two categories (meaning the increase in cases was decelerating) indicated that from a public health perspective, the lockdown had a positive effect on the pandemic. However, the growth rate never fell to below 1.0 immediately following the lockdown, so the lockdown was not sufficient to stop the pandemic, which is borne out by obvious international persistence of this infection and a growing worldwide death rate.^2^ This has caused countries to impose new lockdowns and encourage residents to isolate themselves in their houses.

Nussbaumer-Streit et al. 2020 [12] in a recent review on lockdown demonstrated that this type of measure has had an effective impact at reducing the incidence and mortality of COVID-19 during the current pandemic. This article recommended that along with other public health procedures, lockdown should be enforced at an early stage to prevent the COVID-19 infection from spreading further. The study has shown evidence that lockdown measures are consistently beneficial, with quarantining of people who were exposed to confirmed or suspected cases preventing 44% to 81% of new cases and 31% to 63% deaths, compared to a lack of any lockdown measure. The authors showed that a lockdown may prove helpful in controlling the COVID-19 outbreak. Our study showed that the lockdown was beneficial in decreasing the rate of growth per day of infection, but ultimately insufficient to bring the absolute growth rate down to 1.0 or less which is the point where an epidemic is clearly under control. The concept of a lockdown is theoretically attractive because it minimizes the number of people exposed to contagious patients and therefore fewer people will be susceptible to getting infected [13].

A lockdown may play an important role when vaccination or prophylactic treatment is not possible, as has been the case with COVID-19 pandemic. In this report, we analyzed the impact of 15 days before 15 days during and 15 days after a lockdown on the prevalence of COVID-19 cases in 27 countries. We found that daily cases of COVID-19 and growth factor results show a declined trends 15 days after the lockdown period. The present study findings did not support our hypothesis that lockdown will significantly decrease the number of cases.

Manchein and colleagues 2020 [14] analyzed the growth of the cumulative number of COVID-19 cases from various countries until the last week of March 2020. Their study findings show that soft lockdown approaches are not suitable to flatten the growth curves. They also found that along with social distancing of individuals, the strategy of identifying and isolating infected individuals at daily basis and large levels can help to compress the curve.

In addition, Bensimon and Upshur 2018 [15] and Greenberger 2018 [16] reported that the efficacy of a lockdown is uncertain. The present study findings are consistent with those of Manchein and colleagues 2020 [14], Bensimon and Upshur 2007 [15], and Greenberger, 2018 [16], that lockdowns or quarantines may also need additional supportive measures such as proper information, social distancing, and hygienic measures to eradicate an epidemic.

In few countries there was a positive impact of lockdown to minimize the incidence of SARS-COV-2. But, this is also fact that, in many countries especially the developing countries long-term lockdown was not sustainable as it has various social, psychological and economic impact. Future lockdown policies should adhere to optimizing behavior such as social distancing and mask wearing associated with social and cultural factors that can affect in minimizing the COVID-19 pandemic, because lockdown alone will not be effective if people will not adhere to this policy.

### Study strengths and limitations

This is the first article in the literature, to our knowledge, that has investigated the impact of a lockdown on epidemiological trends of prevalence and mortality of the COVID-19 pandemic, and our findings are based on the twenty seven countries worldwide. During the COVID-19 pandemic, to date, only mathematical modeling-based reviews have been published to hypothesize the impact of a lockdown on the prevalence of COVID-19 cases. This is the first study, which analyzed the impact of 15 days before, 15 days during and 15 days after lockdown on the prevalence trends of COVID-19. Another strength is that the study data were gathered using reliable sources including “World Health Organization, and concerned countries”. We also analyzed the growth factor and the growth rate per day, which are vital metrics to determine the epidemiological trends of a pandemic. A limitation is that we were unable to investigate confounding factors including how much people vary in: (1) adherence to lockdown, (2) adoption of protocols of social distancing, (3) practice of health hygienic conditions and (4) experience disease testing systems of their individual countries.

## Conclusions

The present study findings show that 15 days after lockdown there was no significant decline in the mean prevalence and mean mortality rate due to novel coronavirus SARS-COV 2 compared to 15 days before and 15 days during the lockdown in 27 countries. Whereas daily cases of SARS-COV-2 patients, and the growth factor results declined and the growth rate per day both declined to an impressive negative level in the case of the growth rate per day by the time period of 15 days after the lockdown period, these two metrics of infection spreading did not fall sufficiently to control the pandemic. These findings may be useful for decision-makers who are contemplating further lockdowns to control the spread of the COVID-19 pandemic. Future lockdown policies should adhere to optimizing behavior such as social distancing and mask wearing associated with social and cultural factors that can affect spreading the COVID-19 pandemic, because lockdown alone will not be effective if people will not adhere to this policy.

## Data Availability

After publication data can be shared.
